# Emotional Expression Processing and Depressive Symptomatology: Eye-Tracking Reveals Differential Importance of Lower and Middle Facial Areas of Interest

**DOI:** 10.1155/2020/1049851

**Published:** 2020-01-06

**Authors:** Laurie Hunter, Laralin Roland, Ayesha Ferozpuri

**Affiliations:** Christopher Newport University, Newport News, VA, USA

## Abstract

The current study explored the eye-tracking patterns of individuals with nonclinical levels of depressive symptomatology when processing emotional expressions. Fifty-three college undergraduates were asked to label 80 facial expressions of five emotions (anger, fear, happiness, neutral, and sadness) while an eye-tracker measured visit duration. We argue visit duration provides more detailed information for evaluating which features of the face are used more often for processing emotional faces. Our findings indicated individuals with nonclinical levels of depressive symptomatology process emotional expressions very similarly to individuals with little to no depressive symptoms, with one noteworthy exception. In general, individuals in our study visited the “T” region, lower and middle AOIs (Area of Interest), more often than upper and noncore areas, but the distinction between the lower and middle AOIs appears for happiness only when individuals are higher in depressive symptoms.

## 1. Introduction

ied"?>Emotion recognition and facial processing are crucial abilities necessary for successful daily social interactions. Depression influences how individuals process their social environment, thus impacting social interactions. Studies have explored how depressed individuals process various emotional stimuli by exploring a preference for or focus on one of multiple images. For instance, Sears et al. [1] measured eye movements of depressed participants who were asked to view 4 different images and reported depressed individuals attended longer to depressive images. Social interactions often involve processing individual facial expressions of emotion, rather than a preference for or focus on one of several images, an important issue addressed in the current study.

Previous research relied on various, inconsistent tasks and processing measures to examine the relationship between depression and emotion processing. Few studies [2–4] have assessed areas of interest on the face and what features depressed individuals attend to when processing facial expressions of emotion. To our knowledge, only two studies have explored how depressed individuals process the various areas of an emotional expression using the eye-tracking technology [3, 4], but these researchers did not specifically address whether any particular facial feature (AOI) yielded greater processing time when judging emotional expressions. Both studies indicated emotion recognition accuracy is enhanced for depressed individuals when they focus on multiple features of the face. To better understand the processing of facial expressions of emotion, we assessed visit duration as our measure of processing time in an effort to better explain where the individual gathers information for the emotional stimuli. We also explored AOIs identified as important in emotion recognition [5] to determine whether depressed individuals use AOIs differently than nondepressed individuals.

Research utilizing faces as stimuli and employing a free-viewing task, is limited, and conclusions are contradictory regarding the negative, unpleasant image bias among depressed individuals. Duque and Vazquez [6] researched visual attention in depressed and nondepressed participants with emotional faces. Individuals with depression spent more time on sad faces and less time on happy faces, and longer first fixations toward sad faces only. Similarly, Caseras et al. [7] supported a negative image bias for first fixation duration and total fixation time in unpleasant emotional faces for depressed individuals. Mogg et al. [8] assessed eye movements of individuals with generalized anxiety disorder and individuals with depression while viewing happiness, sadness, anger, and neutral faces. There were no group differences in initial fixations revealing no negative attentional bias. These researchers did not utilize fixation duration, thus limiting conclusions regarding the negative image bias on emotional faces. These inconsistent findings may be explained by the task itself. In the aforementioned studies, participants simply viewed the faces without any instruction to judge emotional content. Perhaps explicit instructions focused on emotion processing which provided a more representative task similar to social interactions, i.e., an emotion judgment task paradigm.

An emotion judgment task paradigm is viewing a stimulus with the assigned task of judging the emotion being displayed. In eye-tracking studies, participants typically view an image or a face on a screen and make an emotional judgment while the researcher tracks the participants' eye movements. We argue this judgment task provides a better understanding of how individuals process emotional interactions.

Only three studies, to our knowledge, have combined a task-oriented paradigm and eye-tracking. Matthews and Antes [2] explored dysphoric and nondepressed participants' eye fixations on emotional images (but not facial expressions). Depressed individuals went to sad images more frequently than nondepressed individuals. Additionally, Schmid et al. [3] explored mood priming on emotion judgment and eye-tracking as well as AOIs on the face. Interfeatural saccade ratio (jumping from feature to feature across the face divided by total jumps) reflected a multiple feature processing approach. The feature gaze duration was used to show single feature processing approach. Schmid et al. [3] concluded when participants were in a sad mood, these two processing styles influenced emotion recognition abilities. Specifically, the multiple feature processing approach was positively related to emotion recognition only when participants were in sadness. Furthermore, the single feature processing approach was negatively related to emotion recognition only when participants were in a state of sadness. There were, however, no findings for happy mood. Furthermore, emotion recognition accuracy was enhanced with depressed individuals taking a multiple AOI approach. Finally, Wu et al. [4] further addressed the issue of features of the face by measuring fixation duration in an emotional expression judgment task with participants who were depressed and not depressed. Their findings indicated, although spending less time on AOIs, depressed individuals were equally as accurate as nondepressed individuals when judging emotional expressions. Of importance to the current study, time spent on the AOIs (the eyebrows, eyes, nose, and mouth) did not differ between the two groups (depressed and nondepressed). The nose AOI yielded the greatest fixation duration compared to the eyes, followed by the mouth, and then the eyebrows. The researchers noted that this finding was likely due to the placement of a fixation cross in the middle of the screen for each trial, an issue which was eliminated in the current study. Furthermore, the current study also addressed noncore features (not the eyebrows, eyes, and nose/mouth) of the face as an AOI, to explore whether those features are utilized in emotion processing.

## 2. Eye-Tracking Metrics

As noted, eye-tracking technology offers a unique way to assess how individuals process emotional stimuli. Typical data processing from eye-tracking studies utilizes fixation metrics which indicate initial interest in facial features but not attentional maintenance on those features, an important construct in emotion recognition [9]. As noted above, some studies focus on initial fixation [1, 2, 6, 8, 10]. Another dependent variable used is fixation duration [2, 4, 6, 7, 10–12]. Initial fixation and fixation duration seem to be the variables of choice, but visit duration may provide more information regarding attentional maintenance because it reflects whether an individual visits and revisits a particular feature of the face. Salvucci and Goldberg [13] explain a visit has a starting point and an ending point similar to a fixation. Fixations are specific points where the individual looks at a given moment in time. Each individual eye movement point serves as a fixation. Fixations, however, do not accurately explain AOIs processing. A visit is when an individual makes an eye movement point on the face, then leaves the point last made to a different area on the face and then returns to the same point initially made. Visit data would better explain significant areas of interest for emotions because it assesses areas used to gather more information, because it represents “a higher-level collection of fixations organized about visual targets and areas” ([13], p. 5).

## 3. Areas of Interest in Emotion Recognition

In emotion recognition literature, research has suggested distinct facial areas (AOIs) express each emotion in a variety of different ways and intensities. In a study conducted by Hasegawa and Unuma [14], participants were shown images which had variation in eyebrow slant, eye opening, and mouth-opening to demonstrate anger and sadness. Anger was more easily recognized when the eyebrows are at a slant, the mouth is open, and the eyes are closed. Sadness was perceived most often when the eyebrows were lowered and the eyes were more closed. The study by Hasegawa and Unuma [14] supports Sullivan's [15] claim stating specific facial areas distinguish specific emotions. Eisenbarth and Alpers [16] also found individuals focused on specific facial AOIs for each emotion, suggesting different parts of the face distinguish specific emotions. For instance, the AOI which is ideal for recognizing happiness may not be the same AOI which is ideal for recognizing another emotion, such as anger. Ekman and Friesen [5] identified three AOIs which assist in emotion recognition: upper, middle, and lower parts of the face. Research exploring AOIs should consider the three areas important for recognition of facial expressions of emotion [5]. The upper AOI includes the eyebrows and forehead, the middle AOI consists of the eyes and cheekbones, and the lower AOI encompasses the nose, mouth, and (Ekman & Friesen, [5] as cited in [15]). Previous research indicated depressive symptomatology impacts how individuals attend to and process facial expression, thus considering specific AOIs when processing emotions warrants further exploration. Wu et al. [4] and Schmid et al. [3] are the only researchers who addressed the AOI regions, but as mentioned earlier, they did not definitively evaluate the role of particular parts of the face in an individual's interpretation of facial expressions of emotion.

In summary, evidence suggests depressed individuals may be drawn to and attend longer to depressive images (either scenes or faces) when asked to view different images. However, in real world social interactions, individuals are not choosing among facial images but rather processing a facial expression to which a response may be necessary, i.e., social interaction. Our limited knowledge of the impact of AOIs on facial emotional processing highlights differences according to various emotions as well as depressive symptoms, but further exploration is necessary. Emotion recognition literature has suggested particular areas of the face are unique for particular emotions, hence, focusing on those areas may enhance processing abilities, and thus it makes intuitive sense to explore how these areas are perceptually and cognitively processed. This process, we argue, is most effectively measured using a less utilized eye-tracking metric, namely visit duration. By definition, visit duration comprises all fixations within a given AOI, for a particular visit. Arguably, visit duration indicates the amount of time an individual spends visiting and returning to the AOI, providing detailed information about how an individual is actively interpreting an emotional expression. The present study expanded on previous literature by further exploring how features of the face (AOIs) may influence emotion processing as a function of depressive symptomatology. We hypothesized individuals with nonclinical levels of depression will utilize AOIs differently than nondepressed individuals and this difference will be impacted by the emotion portrayed.

## 4. Method

### 4.1. Participants

Fifty-three college-aged students (40 women, 13 men, *M*_age_ = 20.75, 72% white) were recruited from a small, liberal arts university in the Mid-Atlantic states through the SONA psychology research participant system.

### 4.2. Procedure and Materials

The procedures were approved by The University's Institutional Review Board. Participants were instructed they would be completing an emotion recognition task while having their eye movements recorded with an eye tracker. Following informed consent and demographic collection, the participants were positioned in front of a 24-inch monitor containing the Tobii X3-120 eye tracking system (Tobii Technology Danderyd, Sweden) fixated to the bottom of the monitor. The Tobii X3-120 eye-tracker analyzes eye movements at 120 Hz per second by utilizing “corneal reflection techniques.” A standard 9-point calibration procedure was used to ensure participants were 67 cm away from the computer screen. After calibration was completed, the participants were shown the emotional label key press they would use to judge each face. The Participants were instructed to judge and label the emotion they thought best represented the emotion present on the displayed face. The label response paradigm involved a keypress with −2 representing anger, −1 for fear, 0 for happiness, 1 for neutral, and 2 for sadness. Four faces were employed for labeling practice. Each face was presented for a total of 4 seconds. Before each face was presented, a fixation cross was shown, in different positions, to direct gaze. The order in which the faces and fixation cross positions were presented was pseudo-randomized. Once participants finished viewing and rating the faces, they were asked to complete the Beck Depression Inventory, a measure of the severity of depression over a two-week period. Participants respond to 21 items using a 4-point severity scale [17]. This widely used assessment of depression symptoms has reported high reliability, *α* = 0.93 [17], high content validity [18], and strong to moderate convergent validity (ranging from 0.58 to 0.79, Richter et al. [18]). None of our participants met the clinical-criteria for depression, but we were able to create a median-split, with half of our participants revealing little to no depressive symptoms (a score of less than or equal to six on the BDI).

### 4.3. Facial Expression Stimuli

Eighty facial expression images of five emotions (happiness, sadness, anger, fear, and neutral) were taken from the Child Affective Facial Expression set, a validated set of child facial expressions [19, 20], and the NimStim set of facial expressions, a valid and reliable set of adult facial expressions [21]. Images were equally distributed across sex, age of face, and emotion of face, yielding 80 images. Images for this study were selected for their highest accuracy rating within each emotion, and all images were free from extraneous factors, such as facial scars, blemishes, or distinctive hair, all images were presented on a solid background, with a white drape covering anything (like clothes) other than the face.

## 5. Results

As we argued earlier, the particular eye-tracking metric analyzed is critical to consider when exploring visual processing during an emotion recognition task. The most commonly used metrics for eye-tracking data are fixation count or fixation duration [22], but we have asserted visit duration which provides more meaningful data for emotional processing. Primary interest is marked by fixation metrics providing information regarding initial processing. Revisiting a particular AOI indicates usefulness or perceived importance for emotion processing, which is represented by the Tobii software variable of visit duration, not fixation duration [9].

A 2 (depression) × 2 (age of face) ×  5 (emotion) × 4 (AOI) mixed model ANOVA was conducted for visit duration only. Several main effects and interactions yielded significance.

The results of these analyses are discussed separately in the sections below. All significant findings are reported, but only those findings relevant to our hypotheses are discussed in terms of follow-up analyses, tables and/or figures. For each significant effect, appropriate follow-up analyses (main effect- LSD, interaction effects- pairwise *t*-tests with Bonferroni corrections) were conducted with the findings discussed below and presented in tables and figures. It is important to note, that the main effects and the two-way interactions are overshadowed by the three-way interaction discussed below. We mention the significance and follow-up analyses here, but conduct further exploratory analyses of the three-way interaction only.

**Figure 1:**
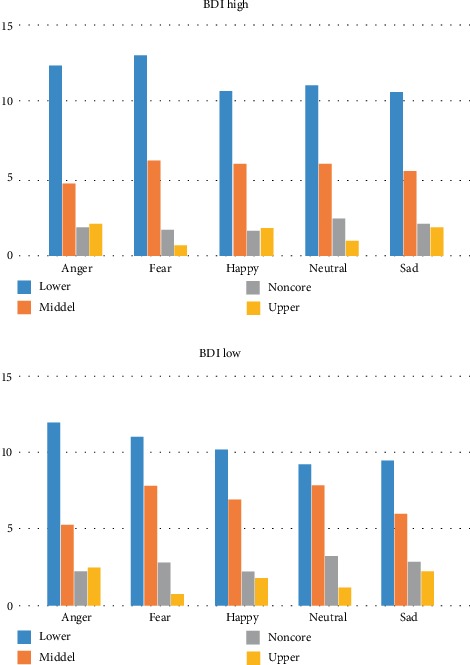
Three-way interaction among depressive symptomatology, emotion, and area of interest.

Consistent with our hypothesis, and of utmost importance for our study, a three-way interaction among emotion, AOI, and BDI yielded significance, *F*(12, 612) = 2.011, *p* = 0.021 (see Figure 1). For each BDI group, low and high, follow-up pairwise comparisons among the AOIs for each emotion with a Bonferroni correction of 0.0008 indicated several significant differences (Table 2). In addition, an age of face, emotion, and AOI interaction yielded significance, *F*(12, 612) = 15.000,*p* < 0.05, as well as an age of face, AOI, and BDI interaction approached significance, *F*(3, 153) = 2.248, *p* = 0.085.

### 5.1. BDI Low

For anger, the lower AOI visit duration was significantly greater than the remaining three AOI's, middle, noncore, and upper. No other differences emerged as significant for anger. For fear, happiness, and neutral, similar patterns emerged, with significant differences in visit duration resulting for all AOIs with the exception of lower compared to middle and noncore compared to upper. For sadness, the lower AOI did not differ from the middle AOI, the middle AOI did not differ from the noncore AOI, and finally, the noncore AOI did not differ from the upper AOI. The lower AOI had significantly greater visit duration than the noncore and upper AOIs; the middle AOI had significantly greater visit duration than the upper AOI.

### 5.2. BDI High

For anger, the lower AOI visit duration was significantly greater than the remaining three AOI's: middle, noncore, and upper. No other difference emerged as significant for anger. For fear, the lower AOI had significantly greater visit duration than the middle AOI, noncore AOI, and the upper AOI; the middle AOI had significantly greater visit duration compared to the noncore and upper AOIs; the noncore and upper AOIs' visit duration did not significantly differ. For happiness, significant differences in visit duration resulted for all AOIs with the exception of lower compared to middle and noncore compared to upper. For neutral, significant differences in visit duration emerged between the lower AOI compared to the noncore AOI and the upper AOI, as well as the middle AOI compared to the upper AOI. All remaining comparisons in visit duration for neutral were not significant. For sadness, the lower AOI visit duration was significantly greater than the remaining three AOI's (middle, noncore, and upper). The middle AOI yielded significantly greater visit duration than the upper AOI, but visit duration was not different between the middle AOI compared to the noncore and upper AOIs.

A main effect for emotion was found, *F*(4, 204) = 12.184, *p* < 0.05. Pairwise comparisons using LSD procedure revealed greater time was spent on fearful faces (*M* = 5.543, *SE* = 0.195), followed by anger (*M* = 5.389,*SE* = 0.189), neutral (*M* = 5.276,*SE* = 0.188), happiness (*M* = 5.9182,*SE* = 0.187), and lastly sadness (*M* = 5.123,*SE* = 0.187). Second, a main effect for AOI was found to be significant,*F*(3, 153) = 98.176, *p* < 0.05. Pairwise comparisons of the means using LSD procedure specified greater amount of time was spent on the lower portion of the face (*M* = 11.010, *SE* = 0.543), followed by middle (*M* = 6.256, *SE* = 0.514), and lastly noncore (*M* = 2.338, *SE* = 0.303) and upper (*M* = 1.605, *SE* = 0.235). No other main effects revealed significance.

An age of face and emotion interaction yielded significance,*F*(4, 204) = 9.015, *p* < 0.05. With adult faces, fear (*M* = 5.854, *SE* = 0.249) revealed significantly greater visit duration compared to anger (*M* = 5.439, *SE* = 0.232), *t*(52) = 4.230, *p* < 0.0025, neutral (*M* = 5.322, *SE* = 0.229), *t*(52) = 4.691, *p* < 0.0025, sadness (*M* = 5.268,*SE* = 0.236), *t*(52) = 6.523, *p* < 0.0025, and happiness (*M* = 5.124, *SE* = 0.226), *t*(52) = 6.883, *p* < 0.0025, which were not significantly different from one another with one exception anger compared to happiness, *t*(52) = 3.430, *p* < 0.0025. But for child faces, only two differences emerged as significant, anger (*M* = 5.360, *SE* = 0.262) compared to sadness (*M* = 4.992, *SE* = 0.261), *t*(52) = 4.406, *p* < 0.0025, and neutral (*M* = 5.255, *SE* = 0.274) compared to sadness, *t*(52) = 3.411, *p* < 0.0025. An age of face and AOI interaction approached significance, *F*(3, 153) = 2.447,*p* < 0.066, indicating for both adult and child faces, greater visit duration for lower and middle AOIs compared to noncore and upper AOIs. An emotion and areas of interest interaction yielded significance,*F*(12, 612) = 19.165, *p* < 0.05. Follow-up pairwise comparisons among the AOIs for each emotion with a Bonferroni correction of 0.00167 indicated several significant differences (See Table 1). For anger, there were significant differences in visit duration among all the AOIs except noncore and upper AOIs. For fear, significant differences emerged in visit duration for all AOIs. For happiness, significant differences in visit duration resulted for all AOIs with the exception of noncore and upper AOIs. For neutral significant differences in visit duration were indicated for all AOIs. Finally, for sadness significant differences emerged in visit duration for all AOIs except noncore to upper AOIs. No other 2-way interactions yielded significance.

## 6. Discussion

The current study offers a new perspective for assessing emotion recognition abilities in individuals with depressive symptomatology by focusing on visit metrics, rather than fixation metrics, from an eye-tracking protocol. We argue visit data encompasses a more representative eye-tracking metric for emotion recognition as it relies on an initial and a follow-up interest as its measure. Our findings indicate individuals with nonclinical levels of depressive symptoms process emotional expressions very similarly to individuals with little to no depressive symptoms, with one noteworthy exception. In general, individuals in our study visited the “T” region, lower and middle AOIs, more often than upper and noncore areas, but the distinction between the lower and middle AOIs appears for all emotions except happiness when individuals are higher in depressive symptoms. In our study, facial expressions were presented as color photographs rather than black and white and as such are more analogous to real-world social interactions thus providing more definitive information regarding emotion recognition processing.

Generally speaking, our findings support the importance of the middle and lower AOIs when processing the emotions of anger, fear, happiness, neutral, and sadness, and also highlight the importance of using multiple facial features to process emotional expressions as noted in previous literature [3, 4]. This use of multiple features, specifically the lower and middle AOIs, reveals a particular pattern we refer to as the “T” which appears to be of utmost importance and critical when processing emotions. In most cases, these depictions indicated very little transitions outside the “T” pattern supporting our findings of less visit durations for noncore and upper AOIs. Thus, when making emotion judgments, participants focus on the eyes, nose, and mouth (lower and middle AOIs), not the eyebrows, forehead, or extraneous features. When examining the patterns, no significant distinctions between the upper AOI and noncore AOI (extraneous features such as hair) are noted, indicating any extraneous features do not lead to greater visit duration for noncore AOI. A unique contribution of the current study is the separation of the eye/middle AOI from the eyebrow/upper AOI, suggesting it is the eyes which contain more emotional information.

The general finding of the “T” pattern is further differentiated when considering depressive symptomatology in a nonclinical population. Specifically, for individuals who have little to no depressive symptoms, eye-tracking patterns for fear, happiness, and neutral follow the aforementioned “T” pattern, with lower and middle AOIs showing similarly high visit durations and upper and noncore AOIs showing similarly low visit durations. Individuals who have higher, yet nonclinical levels of depressive symptoms, display a “T” pattern for happiness only. Thus, all participants, regardless of depressive symptomatology, show the “T” pattern when processing happiness. Perhaps, the uniqueness of happiness explains this phenomenon. First, happiness is uniquely characterized by the smile and after processing the smile, the focus can shift to the next important feature, the eyes, with continued transitioning between those two AOIs. Second, previous literature acknowledges happiness is rather quickly and usually accurately recognized, thus group differences may not be detected in this emotion. In addition, happiness is the only pleasant emotion so more general exploration of important emotional features is warranted. For fear and neutral, nondepressed individuals demonstrate the “T” pattern as well, thus supporting the more generalized exploration pattern of important facial features for these two emotions. Fear and neutral are more complex emotions than anger and sadness, so one may need to disperse attention to multiple features to garner the necessary information to process that emotion. Whereas in anger and sadness, the lower AOI is rather unique and dramatic, sadness displays an extremely pouty mouth, and anger displays exposed teeth, so focus is less likely toward the middle AOI. When depressive symptomatology is higher, however, individuals focus on the lower AOI more than the middle AOI for fear, neutral, anger, and sadness. Perhaps, depressed individuals draw their gaze to the singular feature of the mouth [3] with more unpleasant emotions to avoid emotional displays which may match depressive symptoms, ignoring the importance of the middle AOI. Thus, these individuals may miss out on important emotional cues and hinder social interactions and perhaps exacerbate depression [23]. Future studies should expand on this finding in samples with clinical levels of depression to explore this interpretation. Particularly, our findings can provide the foundation for therapeutic interventions designed to assist populations with mild levels of depression. Individuals with emotional processing deficits may benefit from understanding how to utilize the eyes and mouth regions of the face.

It must be noted the current study evaluates eye-tracking patterns without regards to accuracy, thus, we cannot definitively state whether these processing strategies support accurate recognition. The stimuli chosen for this study were the highest accuracy ratings from the stimuli set, and as such may limit our ability to explore how accuracy is impacted by our patterns. Future studies should not only explore the relationship between areas of interest and accuracy of emotional expression judgment, but also utilize stimuli with lower accuracy ratings to eliminate any potential ceiling effects. Given the universality of emotional expression recognition and the importance of the lower and middle AOIs when visually processing emotional images, we would expect the same pattern of results with stimuli with low separability. Furthermore, each image was presented for four seconds, so comparisons of gaze time for particular emotions is not possible. As such, we cannot address previous literature reporting longer gaze periods for sad images in individuals with depressive symptoms. Future studies could address this issue by removing the set time of image exposure.

The interaction among age of face, AOI, and BDI group approached significance, but the trend is worth noting and is particularly interesting given the own-age/other-age bias emotion recognition literature (e.g., [16]). When young adults are exploring child emotional expression images, a more exploratory technique of using the lower and middle AOIs seems indicative on an other-age bias. This pattern is also noted among depressive symptomatology, but is not statistically meaningful. When judging the emotional expressions of children, college-aged individuals with little to no depressive symptoms utilize the features of the “T” equally to gather critical information to understand the emotion. College-aged individuals with nonclinical levels of depressive symptoms, however, utilize the “T” pattern but with clear preference for the lower AOI, for reasons noted above. Further exploration of this other-age bias with child faces and depressive symptoms appears to be a fruitful area of research, worthy of future study.

In conclusion, we have presented findings which clarify the important areas of interest (facial features) when processing emotional expressions to reflect previous literature describing the roles of various facial features (e.g., [5]). This clarification aligns with a general “T” pattern indicating the importance of the eyes and mouth areas. Further differentiation of these important areas is noted, however, when we consider depressive symptomatology in a nonclinical sample.

## Figures and Tables

**Table 1:** Follow-up *t*-tests for two-way interaction between emotion and AOIs.

Emotion	Lower	Middle	Upper
Anger			
Middle	8.655^∗^		
Noncore	14.932^∗^	4.789^∗^	−0.398
Upper	11.326^∗^	4.831^∗^	

Fear			
Middle	5.418^∗^		
Noncore	14.075^∗^	6.311^∗^	4.012^∗^
Upper	16.269^∗^	11.894^∗^	

Happiness			
Middle	4.454^∗^		
Noncore	15.135^∗^	6.317^∗^	0.467
Upper	14.271^∗^	8.200^∗^	

Neutral			
Middle	3.464^∗^		
Noncore	11.090^∗^	5.366^∗^	3.940∗
Upper	15.288^∗^	10.324^∗^	

Sadness			
Middle	5.623^∗^		
Noncore	13.264^∗^	5.016^∗^	0.816
Upper	10.651^∗^	6.287^∗^	

^∗^significant at the 0.00167, Bonferroni correction.

**Table:** (a) BDI low

Emotion	Lower	Middle	Upper
Anger			
Middle	7.932^∗^		
Noncore	14.355^∗^	3.439	−0.230
Upper	9.630^∗^	3.672	

Fear			
Middle	3.197		
Noncore	10.622^∗^	4.460^∗^	3.667
Upper	14.406^∗^	9.873^∗^	

Happiness			
Middle	2.678		
Noncore	11.743^∗^	4.321^∗^	0.854
Upper	10.466^∗^	6.784^∗^	

Neutral			
Middle	1.382		
Noncore	7.809^∗^	4.142^∗^	3.128
Upper	14.149^∗^	8.734^∗^	

Sadness			
Middle	3.890		
Noncore	11.543^∗^	3.253	0.803
Upper	8.923^∗^	4.365^∗^	

**Table:** (b) BDI high

Emotion	Lower	Middle	Upper
Anger			
Middle	5.007^∗^		
Noncore	8.322^∗^	3.290	−0.334
Upper	6.698^∗^	3.075	

Fear			
Middle	4.591^∗^		
Noncore	10.119^∗^	4.531^∗^	1.878
Upper	9.899^∗^	7.060^∗^	

Happiness			
Middle	3.664		
Noncore	9.888^∗^	4.742^∗^	−0.456
Upper	9.549^∗^	4.739^∗^	

Neutral			
Middle	3.483		
Noncore	8.341^∗^	3.383	2.367
Upper	9.364^∗^	5.943^∗^	

Sadness			
Middle	4.096^∗^		
Noncore	8.441^∗^	4.014	0.035
Upper	6.631^∗^	4.572^∗^	

^∗^Significant at the 0.0008, Bonferroni correction.

## Data Availability

The eye-tracking and demographic data used to support the findings of this study are available from the corresponding author upon request.
